# From MRD To Match: the Role of Allogeneic Hematopoietic Cell Transplant in Philadelphia-Negative B-ALL

**DOI:** 10.1007/s11899-025-00760-3

**Published:** 2025-11-04

**Authors:** Jessica El-Asmar, John C. Molina, Betty Ky Hamilton

**Affiliations:** 1 Department of Hematology and Medical Oncology, Taussig Cancer Institute, Cleveland Clinic, Cleveland, OH USA; 2 Leukemia Program, Department of Hematology and Medical Oncology, Taussig Cancer Institute, Cleveland Clinic, Cleveland, OH USA; 3 Blood and Marrow Transplant Program, Department of Hematology and Medical Oncology, Cleveland Clinic Cancer Institute, 9500 Euclid Avenue CA-60, Cleveland, OH 44195 USA

**Keywords:** Philadelphia-negative acute lymphoblastic leukemia, B-cell acute lymphoblastic leukemia, Allogeneic, Hematopoietic cell transplant, Cellular therapies, Measurable residual disease

## Abstract

**Purpose of Review:**

Given the high risk of relapse for Philadelphia-negative (Ph-negative) B-cell acute lymphoblastic leukemia (ALL), allogeneic hematopoietic cell transplantation (allo-HCT) is often recommended following first complete remission (CR1) in high-risk patients. However, in the era of measurable residual disease (MRD) testing, allo-HCT may not be indicated for patients with standard-risk disease. Here we review the use of allo-HCT and other consolidative approaches for standard- and high-risk Ph-negative ALL, based on MRD following induction therapy.

**Recent Findings:**

Allo-HCT is strongly indicated for patients with high-risk Ph-negative ALL, especially those who are MRD positive at end of induction. Ongoing trials using cellular and immune therapies such as blinatumomab, inotuzumab ozogamicin, and chimeric antigen receptor (CAR) T-cell therapies have shown promising results in deepening response and decreasing relapse. Further, these agents have demonstrated overall manageable safety profiles.

**Summary:**

The role for allo-HCT following CR1 in patients with standard risk Ph-negative ALL is evolving with advances in therapeutic approaches. MRD is emerging as a critical prognostic factor regardless of treatment strategy, thus questioning the necessity of transplant in MRD-negative patients. With the advances in safety and accessibility of allo-HCT as well as novel therapeutics, overall outcomes in ALL continue to improve.

## Introduction

Acute lymphoblastic leukemia (ALL) is a hematologic malignancy arising from lymphoid precursors that acquire genetic aberrations leading to alterations in cell cycle checkpoint regulation and downstream immature lymphoid clonal proliferation. With over 30 prognostically-relevant subtypes of ALL, the genomic heterogeneity of this disease has made therapeutic approaches challenging. Over the past two decades, our understanding of the molecular basis of ALL has advanced, revolutionizing treatment paradigms and improving patient outcomes.

The typical approach to front-line treatment of ALL in both pediatric and adult populations is multi-agent chemotherapy regimens, with the exception of frail or elderly patients who are not medically fit to receive intensive induction chemotherapy. Ph-negative ALL is a molecular subtype that lacks the *BCR::ABL1* rearrangement or chromosomal t(9;22). More than 70% of patients with Ph-negative ALL will achieve a morphologic complete remission with the standard chemotherapy agents upfront, however many patients will remain at risk for relapse. High-risk cytogenetic and molecular abnormalities, central nervous system involvement, and refractory disease after induction therapy, can indicate increased risk for relapse [1]. Positive measurable residual disease (MRD) at the end of induction therapy is also one of the high-risk features in Ph-negative ALL that would indicate a higher relapse rate. In patients with Ph-negative ALL who achieve first complete remission (CR1) but have persistent MRD, chemotherapy alone will not achieve a deep remission. Allogeneic hematopoietic cell transplantation (allo-HCT) thus plays a key role in the treatment of these patients with high-risk Ph-negative ALL in CR1 [2].

Consolidation with allo-HCT has been shown to reduce relapse risk and improve disease-free survival (DFS), with a trend towards increased overall survival (OS) in patients with high-risk Ph-negative ALL [3]. Allo-HCT is also a recommended treatment option for patients with ALL who relapsed or have refractory disease, regardless of risk stratification [3]. However, the decision to consolidate with allo-HCT in patients with Ph-negative ALL without high-risk features is not as clearly outlined. Therefore, treatment approaches are evolving based on response to post-induction therapy for patients with standard-risk ALL. Novel therapies are also shifting the treatment landscape in select patients, offering alternative approaches in order to achieve more durable remissions and reduce the need for allo-HCT. The purpose of this review is to evaluate the role of allo-HCT in the era of MRD testing among patients with Ph-negative ALL.

**Fig. 1:**
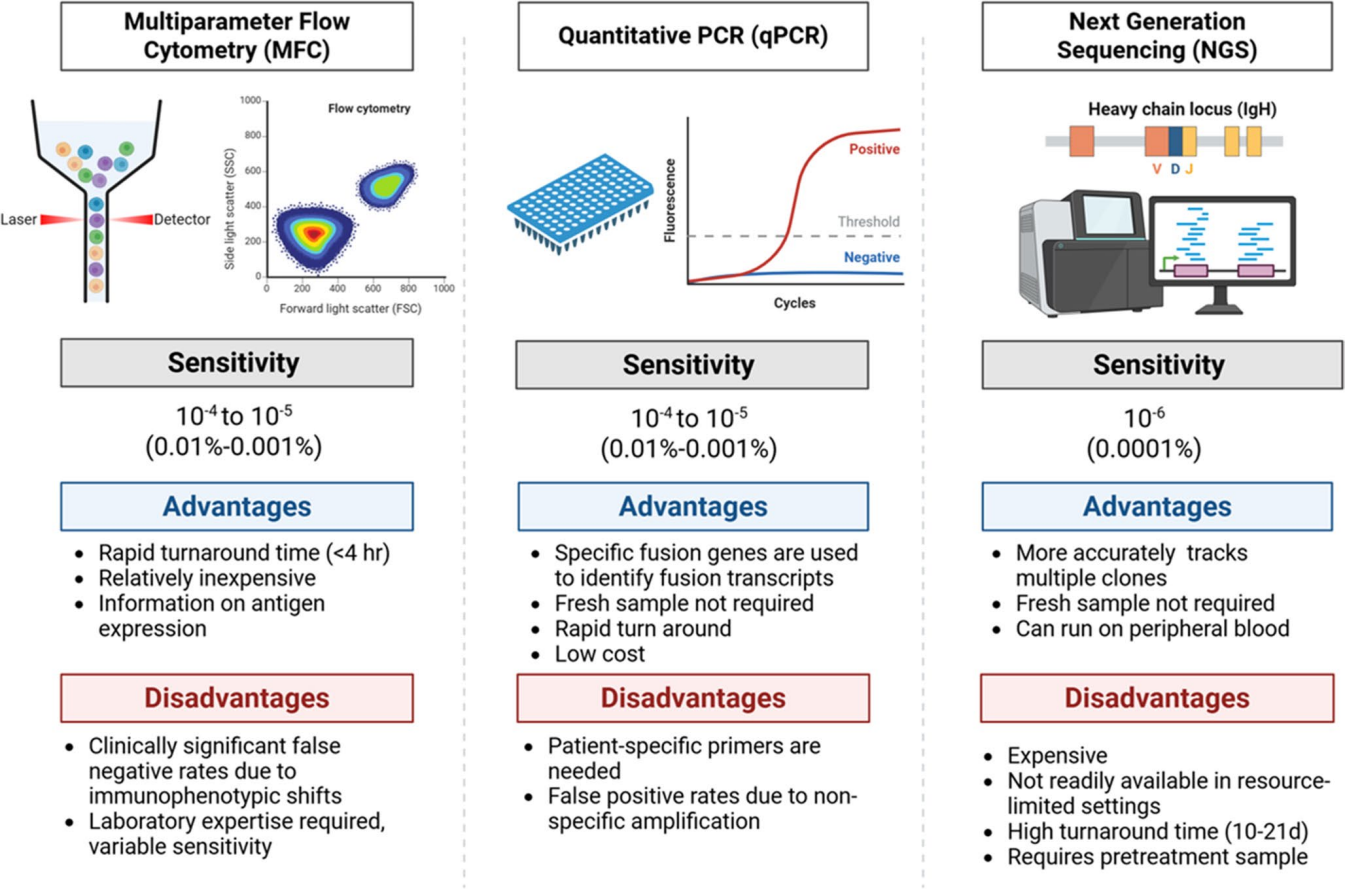
Comparison of MRD assays in ALL

**Fig. 2:**
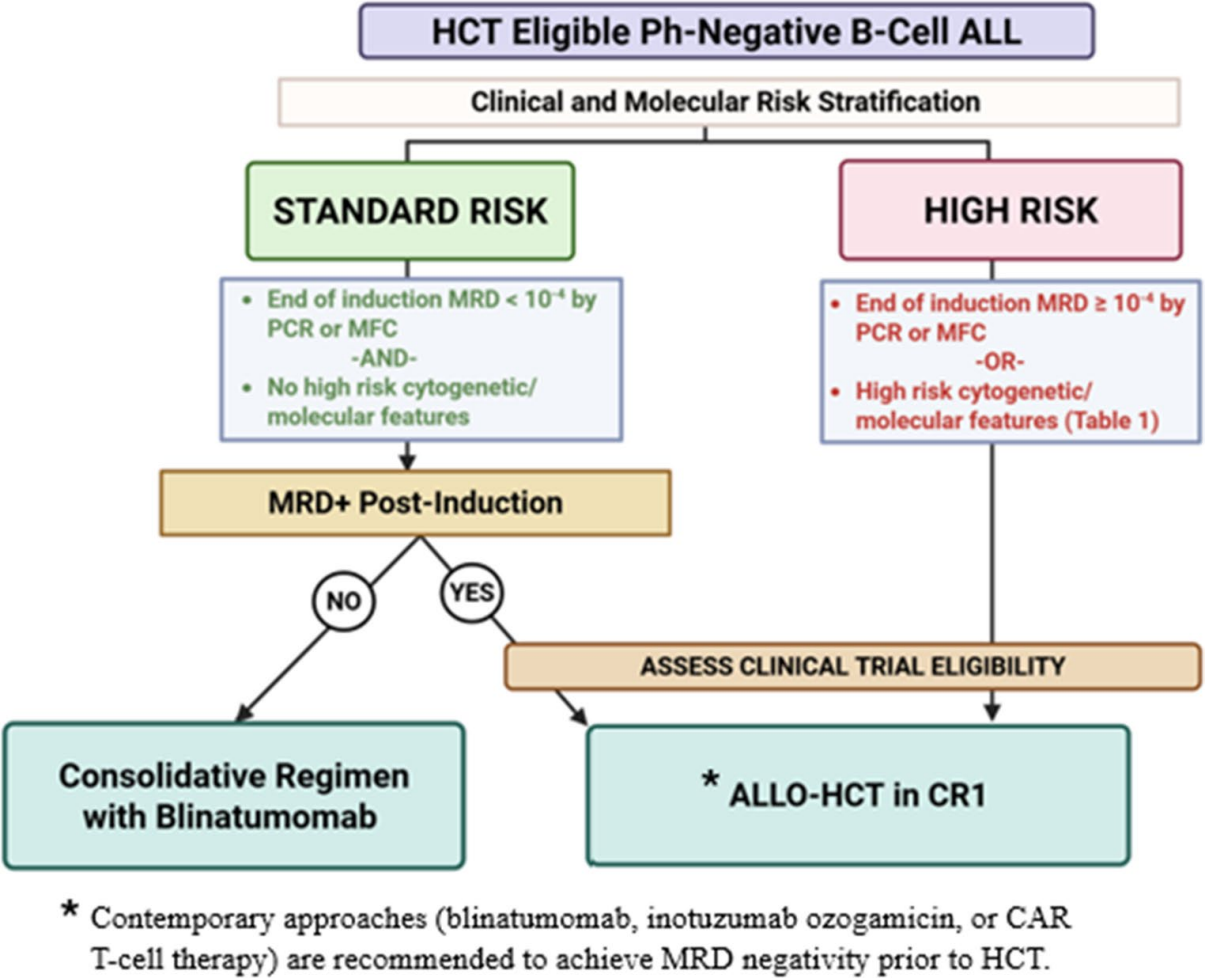
Treatment considerations for Ph-negative ALL based on risk stratification

## Subtypes of Ph-negative ALL

The diagnostic approach to ALL starts with morphologic evaluation of the bone marrow and blasts, and increasingly important, with genomic analysis. Comprehensive identification of ALL subtypes is generally identified via immunophenotyping defined by flow cytometry or immunohistochemistry, cytogenetics, and Next-Generation Sequencing (NGS). Additional studies include detection of cryptic rearrangements via fluorescence in situ hybridization (FISH) and reverse transcription polymerase chain reaction (RT-PCR).

Although Ph-negative ALL in and of itself is not classified as a high-risk subtype of ALL, it is still often associated with poor outcomes. Several clinical, cytogenetic, and molecular high-risk features have been identified, including: elevated white blood cell count >30 × 10^9^/L at diagnosis, age above 40 years, as well as persistent MRD and prolonged time to achieving CR1 (usually longer than 4 weeks) or requiring several induction cycles to achieve CR1 [4]. High risk cytogenetic and molecular abnormalities are detailed in Table 1 and include: *KMT2A* rearrangements, *IKZF1* gene alterations, hypodiploidy (< 44 chromosomes), complex karyotype, or Ph-like gene signatures which include translocations involving *CRLF2* [5]

**Table 1:** Summary of high-risk features of Ph-negative B-cell ALL

**Cytogenetics**
• -7 • +8 • t(4;11) • t(8;14) or t(8;22) • Intrachromosomal amplification of chromosome 2 (iAMP 21) • Complex karyotype (5 or more chromosomal aberrations) • Low hypodiploid (32-39 chromosomes) • Near haploid (24-31 chromosomes)
**Molecular**
• *CRLF2 *rearrangement • *KMT2A *(MLL) rearrangement • *IKZF1 *deletion or loss-of-function mutations • *TP53 *mutation • *JAK2 *mutation • *IDH1/2 *mutations
**Measurable Residual Disease (MRD)**
• End of induction MRD ≥10^-4^ by PCR or MFC
**Clinical Features**
• Age ≥ 40yo in adults • Presenting white blood cell count ≥ 30 x10^9^ /L • Central nervous system involvement • Time to CR1 > 4 weeks • Requiring > 1 induction cycle • Relapse beyond CR1

Several multi-institutional trial protocols have been created by research organizations focusing on ALL treatments. These major groups include the United Kingdom Acute Lymphoblastic Leukemia group (UKALL), Northern Italy Leukemia Group for Acute Lymphoblastic Leukemia (NILG-ALL), Gruppo Italiano Malattie EMatologiche dell’Adulto Leucemia Acuta Linfoblastica (GIMEMA-LAL), and the Spanish group Programa Español de Tratamientos en Hematologia (PETHEMA). Data on over 700 patients from UKALL14, NILG-ALL10/07, GIMEMA-LAL1913, and PETHEMA-ALL-HR2011 demonstrate that patients with high-risk features had increased rates of relapse (hazard ratio 1.85 to 3.28) and death (hazard ratio 1.73 to 3.03) compared to those with standard-risk [6]. Several analyses have demonstrated improved DFS among patients with high-risk ALL in CR1 who undergo allo-HCT [1, 7].

## Clinical Use of MRD

In patients with Ph-negative ALL, MRD has become an important prognostic factor for survival outcomes [8]. MRD persistence has been associated with an increased risk of relapse [9]. Up to 40% of patients who are MRD negative by NGS or flow cytometric immunophenotyping are also still at risk for disease relapse [10, 11], which raises the question of the sensitivity of the MRD assays used and how to determine at which level patients would be at risk for relapse.

MRD may be assessed by multiparameter flow cytometry (MFC), quantitative polymerase chain reaction (qPCR), or NGS (Figure 1). Sensitivity of standard multiparameter flow cytometry MRD is in the order of 10^−4^ or even 10^−5^. Assays using qPCR technologies require fusion genes to serve as unique markers for identifying fusion transcripts. qPCR can detect MRD at a sensitivity 1 × 10^−5^ [12]. NGS assays are capable of detecting residual leukemic cells in 1 out of 1,000,000 (sensitivity of 1 × 10^−6^) [13]. While the use of MFC to detect MRD status has more rapid turn-around time and is relatively inexpensive compared to NGS assays, MFC relies on immunophenotyping, which can lead to variable sensitivity and false negative results, and therefore requires review by a highly trained and experienced hematopathologist with expertise in MRD assessment for ALL [14]. Highly sensitive MRD detection assays via NGS technologies are being increasingly utilized to provide more accurate prognostic data.

Several studies have demonstrated the prognostic impact of end-of-induction MRD testing among adults with B-cell ALL [15]. In a phase II study (CALGB 10403), MRD detection by MFC following induction chemotherapy was shown to be an important prognostic factor for outcomes such as OS and DFS in patients with ALL [15]. A systematic review and meta-analysis of over 20 studies by Bassan et al. confirms superior relapse-free survival (RFS) (hazard ratio 2.34) and OS (hazard ratio 2.19) among patients who achieve MRD negativity, independent of MRD detection method [16]. Given higher risk of relapse, this highlights the potential role for allo-HCT in patients with MRD positivity post-induction.

A few studies have investigated the use of MRD in clinical decision-making. The Spanish PETHEMA group investigated post-remission therapy for adults, age 18–60 years, with high-risk Ph-negative ALL based on MRD levels by MFC. MRD positive patients were assigned to allo-HCT, and those who were MRD negative received further chemotherapy and maintenance [17]. In PETHEMA ALL-AR03, patients who were MRD positive and underwent allo-HCT (*n* = 71) had a 5-year DFS and OS of 32% [95% CI 19% to 45%] and 37% [95% CI, 24% to 50%] compared to 55% [95% CI, 45% to 65%] and 59% [95% CI, 47% to 71%] in MRD negative patients receiving chemotherapy (*n* = 108), respectively [17]. A second study, PETHEMA ALL-HR-11, used more stringent definitions of high-risk ALL as well as standardized and more sensitive MRD testing; and again demonstrated inferior 5-year OS in the allo-HCT group compared to the chemotherapy group (38% vs. 59%, *p* < 0.001) [18]. Although these studies do not directly compare outcomes between chemotherapy and transplant among MRD-negative or positive patients, it does highlight that poor MRD clearance is a prognostic factor for both inferior OS and DFS [17].

## Consolidation Allo-HCT

While allo-HCT offers the potential for long-term remission for patients with high-risk Ph-negative ALL, the procedure is associated with considerable toxicities and mortality rates [19]. Thus, for patients with standard-risk ALL who are MRD negative in CR1, it is less clear if consolidative allo-HCT should be offered to deepen the remission [7]. The benefit of reducing relapse risk must be balanced against transplant-related morbidity and mortality among adult patients with standard-risk Ph-negative ALL. A summary of allo-HCT outcomes in Ph-negative ALL can be seen in Table 2.

**Table 2:** Outcomes of allo-HCT among patients with Ph-negative ALL in CR1

**Study**	**Total Number of Patients**	**Number of Patients in CR1**	**Risk Stratification**	**Overall Survival (OS)**	**Clinically Relevant Outcomes**	**Comparative Arms**	**MRD**
**Huguet et al.** [24]	225	Not specified	Clinical and cytogenetic features	42-month OS: 60%	42-month EFS: 55%	Pediatric-Inspired Regimen	Not reported
**Dhédin et al.** [23]	522	282	Clinical features and post-induction MRD	Not specified	3-year RFS: 64.7% in allo-HCT arm NRM: 15.5%	Chemotherapy vs allo-HCT	≥ 10^-3^ by qPCR
**Zhang et al. **[15]	97	97	MRD status at CR1	3-year OS: 81.8%	3-year LFS: 81.8%	Chemotherapy vs allo-HCT	10^-4^ to 10^-3^ by MFC
**Lv et al. **[20]	131	114	Clinical features, cytogenetics, MRD	2-year OS: 91.2%	2-year LFS: 80.9%	Chemotherapy vs haploidentical allo-HCT	10^-4^ to 10^-3^ by MFC

The rationale behind the success of allo-HCT has been the graft-versus-leukemia effect. Several studies have investigated the optimal conditioning regimen for patients with ALL undergoing allo-HCT [20]. The phase III multicenter study, FORUM, compared total body irradiation (TBI)-based conditioning to chemo-conditioning (using fludarabine, thiotepa, and either treosulfan or busulfan) among a total of 413 patients with high-risk ALL randomly assigned in a 1:1 fashion [21]. All study arms used myeloablative doses of TBI or chemo-conditioning in this study design. Compared to chemo-conditioning, the group that received TBI-based conditioning had statistically significant superior 2-year OS: 0.91 [95% CI 0.86 to 0.95] following TBI vs. 0.75 [95% CI 0.67 to 0.81] following chemo-conditioning [21]. TBI-based conditioning was also associated with improved 2-year cumulative incidence of relapse [0.12, 95% CI 0.08 to 0.17] and treatment-related mortality [0.02, 95% CI < 0.01 to 0.05] compared to chemo-conditioning [0.33, 95% CI 0.25 to 0.40; 0.09, 95% CI 0.05 to 0.14], respectively [21]. Simms et al. also described improved outcomes with TBI-based conditioning compared to chemo-conditioning [22]. 43 patients received etoposide with either TBI or busulfan [22]. 3-year event-free survival (EFS) was 58% in the TBI group compared to 29% to the non-TBI group (*p* = 0.03) [22]. There was a trend towards improved 3-year OS for patients in the TBI arm at 67% compared to 47% in the Bu arm, however this was not statistically significant (*p* = 0.09) [22].

The Group for Research on Adult Acute Lymphoblastic Leukemia (GRAALL) conducted trials between 2003 and 2011 to evaluate the role of allo-HCT among patients with Ph-negative ALL who were treated with a pediatric-inspired chemotherapy protocol. In the GRAALL-2003 trial, 65 of 282 patients received allo-HCT [23]. At 3 years post-transplant, RFS was 64.7% [95% CI, 59% to 70%] and OS was 69.5% [95% CI, 63% to 75%] [23]. Patients with high-risk Ph-negative ALL who did not receive allo-HCT in CR1 with post-induction MRD detection experienced inferior RFS [23, 24]. Conversely, patients with standard-risk disease had improved DFS without consolidative transplantation [23, 24]. Patients without poor-risk molecular features appeared to have no benefit from allo-HCT in terms of RFS and OS, regardless of MRD status [23].

## Novel Therapies

Patients who are older or unfit for allo-HCT historically have a dismal prognosis due to limited therapeutic options. Novel therapies such as bispecific T-cell engagers (BiTE), antibody drug conjugates (ADC), and chimeric antigen receptor (CAR) T-cell therapies have demonstrated promising reductions in morbidity and mortality in the older or more frail, as well as improved efficacy in eradicating MRD. These novel B-cell antigen-directed therapies are also being increasingly used for patients in CR1 with standard-risk Ph-negative ALL who would otherwise not have received allo-HCT, or for patients with relapsed disease regardless or risk group. The advantage of these novel therapies is their limited toxicity compared to allo-HCT, in addition to their impressive remission rates, therefore representing excellent treatment alternatives to intensive consolidative approaches. This treatment approach is especially important when evaluating MRD status as part of post-induction relapse risk assessment.

### Blinatumomab

Blinatumomab, a BiTE therapy that targets CD19 expressed on ALL blasts as well as CD3 expressed on immune T-cells, is a promising therapy in patients with Ph-negative ALL, regardless of risk group. After its approval in the relapsed/refractory setting, blinatumomab was evaluated in the frontline setting as part of the induction phase for patients with Ph-negative ALL per SWOG 1318 [25]. A phase II trial from MD Anderson Cancer Center also evaluated the benefit of blinatumomab consolidation, showing 3-year RFS at 73% [95% CI, 56 to 85] [26]. The use of blinatumomab upfront has shown remission and survival benefits in patients with ALL, regardless of MRD status [27]. The data from E1910, a phase III trial, supported the use of blinatumomab among patients with ALL who are MRD negative post-induction therapy [28]. The trial included over 200 adult patients with Ph-negative ALL who achieved MRD negativity by MFC following induction and were randomized to receive chemotherapy and blinatumomab vs. chemotherapy alone [28]. Three-year OS was superior in the blinatumomab group compared to the chemotherapy only group (85% and 68%, *p* = 0.002), respectively [28].

In young adults with Ph-negative B-cell ALL, blinatumomab has been explored in the frontline setting with cytotoxic chemotherapy. Chiaretti et al. found that adding blinatumomab to the chemotherapy backbone achieved MRD negativity by qPCR in 93% of patients with a DFS of 66% and OS of 71% at 37.5 months [29]. In the HOVON phase II trial, blinatumomab combined with conventional chemotherapy also resulted in improved CR rates and MRD negativity by qPCR post-induction [30]. Jabbour et al. show that the use of blinatumomab with hyper-CVAD (hyperfractionated cyclophosphamide, vincristine, doxorubicin, dexamethasone alternating with high dose methotrexate and cytarabine) achieved a 3-year RFS of 73% without allo-HCT [26].

Collectively, these studies demonstrate promising results for the use of BiTE therapy in the upfront setting in patients with newly diagnosed Ph-negative B-cell ALL, including high-risk disease. The addition of blinatumomab in the induction phase leading to improved MRD negativity may help decrease the need for allo-HCT and its associated complications. Gökbuget et al. evaluated survival data for 110 patients with B-cell ALL who received blinatumomab, including 74 patients who received allo-HCT in CR1 with negative MRD by qPCR after blinatumomab [31]. Their data suggests similar longer-term OS between the group of patients that received allo-HCT compared to those who did not [31].

### Inotuzumab Ozogamicin

Inotuzumab ozogamicin (InO) is an ADC composed of a humanized CD22-directed IgG4 monoclonal antibody (inotuzumab), a cytotoxic agent that leads to DNA cleavage (calicheamicin), and a linker that binds calicheamicin to inotuzumab. Once the complex binds to CD22 expressed on B-cell blasts, it is internalized, and calicheamicin is then released to cause breaks in the double-stranded DNA structure, leading to cell death [32]. InO is approved for use in the relapsed or refractory setting for adult patients with Ph-negative B-cell precursor ALL based on the INO-VATE ALL trial [32]. This phase III trial randomized over 300 patients with relapsed or refractory ALL to InO or standard of care [32]. Patients in the InO group achieved significantly higher rates of complete remission at 80.7% [95% CI, 72.1 to 87.7] compared to 29.4% [95% CI, 21.0 to 38.8] in the chemotherapy arm [32]. Additionally, progression-free survival was superior in the InO arm at 5 months [95% CI, 0.31 to 0.96] compared to 3.1 months among those who received chemotherapy [95%CI, 1.4 to 4.9] [32]. MRD negativity rates detected by MFC were higher among those who received InO compared to standard chemotherapy (78.4% and 28.1%, respectively) [32].

The German Multicenter Study Group for Adult ALL (GMALL) examined the use of InO with dexamethasone alone for older patients with CD22-positive Ph-negative B-cell ALL as part of the INITIAL-1 study [33]. In this phase II trial, patients were older than 55 years of age [33]. All 43 patients achieved CR, with more than 70% achieving negative MRD by real-time qPCR after induction therapy with InO [33]. OS was 91% at 1 year and 73% at 3 years, while EFS was 88% at 1 year and 55% at 3 years [33].

EWALL-INO is a phase II trial investigating the use of InO in addition to a chemotherapy-based regimen CVD (cyclophosphamide, vincristine, dexamethasone) in the frontline setting for 131 patients with CD22-positive Ph-negative B-cell ALL (whether high-risk or standard-risk) who are 55 years or older [34]. 90% of patients who received three doses of InO achieved a CR, and 80% had undetectable MRD by MFC [34]. A phase I/II trial by Jabbour et al. at MD Anderson Cancer Center found that adding InO to CVD achieved 99% overall response rate and 94% MRD negativity by MFC among 80 patients over the age of 60 years [35]. InO is therefore considered safe and effective in the frontline setting for patients with ALL regardless of risk group, namely for older patients who may not tolerate intensive cytotoxic chemotherapy-based induction regimens.

Sinusoidal obstruction syndrome/veno-occlusive disease (SOS/VOD) is a risk associated with InO. The risk is particularly high in patients who pursue allo-HCT following InO. Kayser et al. performed a retrospective study on 58 patients with relapsed or refractory ALL who had received InO followed by allo-HCT [36]. 29% of patients developed SOS/VOD, of which 53% died not in relapse [36]. Interestingly, number of InO cycles and time from last InO administration preceding allo-HCT did not have a statistically significant impact, while conditioning with double alkylator regimen did [36]. This highlights the need to recognize SOS/VOD risk factors as treatment with InO is an effective bridge to allo-HCT.

### Chimeric Antigen Receptor T-cell Therapy

There are three CD19-targeted CAR T-cell therapies that are FDA approved for relapsed B-cell ALL: brexucabtagene autoleucel (brexu-cel), tisagenlecleucel (tisa-cel), and obecabtagene autoleucel (obe-cel) [37, 38]. A summary of the available CAR T-cell products for ALL can be found in Table 3. Brexu-cel is approved for adults aged 26 years or older with relapsed/refractory B-cell ALL [39, 40]. Among the 78 patients in the phase I/II ZUMA-3 trial who received brexu-cel, 60% achieved a CR with complete hematologic recovery, median RFS was 11.6 months [95% CI, 2.7 to 20.5], and median OS was 25.4 months [95% CI, 16.2 to not estimable] [39]. Grade 3 or higher cytokine release syndrome (CRS) and immune effector cell-associated neurotoxicity (ICANS) occurred in 24% and 25% of patients who received brexu-cel, respectively [39].

**Table 3:** CAR T-cell products for ALL

	**Target Antigen**	**Co-Stimulatory Domain**	**FDA Approval (year)**	**Pivotal Trial (NCT)**	**Clinical Indication**	**Complete Response Rates**	**Grade ≥3 CRS (%)**	**Grade ≥3 ICANS (%)**	**Grade ≥3 Hematologic Toxicities**
**Brexucabtagene autoleucel (41)**	CD19	CD28	2021	ZUMA-3 (NCT02614066)	R/R B-ALL ≥18yo ≥1 prior lines of therapy	56-73%	24-26%	25-35%	• Neutropenia (90%) • Anemia (70%) • Thrombocytopenia (80%)
**Tisagenlecleucel (42)**	CD19	4-1BB	2017	ELIANA (NCT02435849)	R/R B-ALL ≤25yo ≥2 prior lines of therapy	60-81%	35-47%	13%	• Neutropenia (90%) • Anemia (70%) • Thrombocytopenia (80%)
**Obecabtagene autoleucel (39)**	CD19	4-1BB	2024	FELIX (NCT04404660)	R/R B-ALL ≥18yo ≥1 prior lines of therapy	55-77%	2-3%	7%	• Neutropenia (80%) • Anemia (60%) • Thrombocytopenia (70%)

Tisa-cel is approved for patients 25 years of age or younger with B-cell ALL in the refractory setting or second or later relapse [37]. Among the 75 patients in the phase I/IIa ELIANA trial who received tisa-cel, 60% achieved a CR with complete hematologic recovery, rate of RFS was 80% [95% CI, 65 to 89] at 6 months and 59% [95% CI, 41 to 73] at 12 months, and rate of OS was 90% [95% CI, 81 to 95] at 6 months and 76% [95% CI, 63 to 86] at 12 months [41]. Grade 3 or higher CRS and ICANS occurred in 35% and 13% of patients who received tisa-cel, respectively [41].

Obe-cel is approved for adults aged 26 years or older with B-cell ALL [37]. Among the 127 patients in the phase Ib/II FELIX study who received obe-cel, 55% achieved a CR with complete hematologic recovery, median EFS was 11.9 months [95% CI, 8.0 to 22.1], and the median OS was 15.6 months [95% CI, 12.9 to not evaluable] [38]. Grade 3 or higher CRS and ICANS was observed in 2.4% and 7.1% of patients who received obe-cel, respectively [38].

While allo-HCT offers the potential for cure, CAR T-cell therapy is not yet considered a definitive cure in this regard. CAR T-cell therapy may offer durable remissions in some instances, however resistance and relapse may still occur through CD19 antigen loss or limited CAR T-cell persistence. Poor response to CD19-targeted CAR T-cell products is postulated to occur via a wide array of mechanisms, including acquired mutations in exons 2 through 5 of the gene which encodes for the CD19 protein, leading to loss of function or allelic silencing [42]. Additionally, studies have looked into the impact of certain factors on the persistence and durability of CD19 CAR T-cell products, including the use of 4-1BB costimulatory domain, the combination of fludarabine with cyclophosphamide as a lymphodepleting regimen, and various antigen escape mechanisms [43]. Recovery of B-cell aplasia is another marker of waning CAR T-cell cytotoxicity against CD19-expressing malignant clones [44]. Collectively, these factors may subsequently lead to suboptimal durability of CAR T-cell therapy and therefore increase the risk for relapses. Currently, CAR T-cell therapy may be used as a bridge to allo-HCT, particularly in patients with ALL who have high-risk features or positive MRD, in order to improve disease-free survival, but the risks and benefits of HCT must be weighed on a case by case basis.

To date, there have not been trials to compare allo-HCT to CAR T-cell therapy in the post-induction MRD positive state upfront. Optimal selection or sequencing between these modalities remains unclear. CAR T-cell therapy following allo-HCT does not appear to increase treatment-related mortality [45]. Feng et al. (2023) show that CAR T-cell therapy after relapse in patients who received allo-HCT is safe and effective, without an increased risk for cytokine release syndrome or graft-versus-host disease [46]. Their study shows 77% of patients achieved CR four weeks following CAR T-cell infusion, with 73% achieving MRD negative status by MFC [46]. There is also ongoing investigation to clarify the role for consolidative allo-HCT following CAR T-cell therapy to achieve durable response. A phase I trial looked at 50 patients with relapsed or refractory B-cell ALL who received CD19-directed CAR T-cell [47]. 31 patients achieved a CR, of which 28 achieved MRD-negativity by flow cytometry [47]. 75% of patients who achieved MRD negativity received allo-HCT: cumulative incidence of relapse rates at 2 years was 9.5% [95% CI, 1.5 to 26.8], 5-year EFS was approximately 62% [95% CI, 38.1 to 78.8], and median OS was over 70 months [95% CI, 10.4 months to not estimable] [47]. However, the concern with performing sequential CAR T-cell therapy followed by allo-HCT would be obliteration of functional CAR T-cells, thus eliminating the benefit of ongoing tumor cell destruction and theoretically affecting durable remission rates [43].

## Future Directions

Figure 2 depicts our proposed treatment algorithm in Ph-negative B cell ALL. MRD has become a significant predictor of relapse in ALL. For otherwise standard-risk Ph-negative ALL patients who achieve MRD negativity post-induction, prognosis is favorable with excellent long-term EFS and OS. These patients generally do not require consolidative allo-HCT as the risks may outweigh the benefits. The use of novel therapies have helped with deepening early responses and have possibly allowed for avoiding transplant. Among patients with ALL who have persistent MRD in CR1, we have convincing data that allo-HCT has shown superior relapse-free survival and disease-related outcomes compared to conventional chemotherapy. However, more studies are needed in this group of patients to compare outcomes among those who receive allo-HCT versus immunocellular therapies.

Efforts to investigate novel therapeutic approaches for patients with standard-risk Ph-negative ALL should continue, with the goal of significantly improving response rates and survival outcomes while minimizing toxicities. The use of B-cell antigen-directed therapies also must be considered in the context of cost-effectiveness analyses. Including these agents in the treatment algorithm to achieve high rates of durable remissions, without the need for allo-HCT, would increase their value in resource-limited settings. It is crucial to focus on accessibility to novel agents through enhanced resource allocation, in order to improve patient outcomes, reduce health care disparities, and bridge the gap between resource-rich and limited settings.

## Conclusion

Survival outcomes and relapse rates for adult patients with Ph-negative ALL are strongly impacted by risk group and measurable residual disease following induction therapy. Due to high risk of relapse post-induction, allo-HCT has been the standard of care for patients with Ph-negative ALL who have high-risk features, and now, who are MRD positive [18]. However, it is important to consider the need for an individualized approach, as the risks associated with allo-HCT may outweigh the benefits, especially in the era of novel therapies. With the advancements in targeted and immune-therapies, it may not be surprising to see deeper remissions with these alternate consolidative regimens. For patients with standard-risk ALL, assessing MRD status in the post-induction phase has become a significant prognostic factor for predicting short-term and longer-term relapse. MRD testing can impact treatment stratification and provide guidance on the need for allo-HCT as consolidation therapy in this subset of patients. Therefore, in the modern era of molecular diagnostics, morphological remission alone is not enough to outline the decision for consolidation therapy.

Whether allo-HCT should be common practice for all patients with standard-risk Ph-negative ALL in CR1 is still an area of ongoing research. MRD response is crucial to informing decisions for allo-HCT in these patients. High sensitivity MRD testing is an invaluable tool to determining next steps in treatment post-induction, and to improve patient outcomes by achieving durable remissions. It is important to use ultra-sensitive MRD assessments to individualize treatment plans based on relapse risk.

In summary, for patients with Ph-negative ALL who achieve CR1, we favor consideration of allo-HCT in patients with MRD positivity after induction. For patients with standard-risk MRD-negative CR1 evaluated via ultrasensitive assays, one can consider non-allo-HCT approaches. These recommendations focus on the importance of individualizing treatment approaches for patients with ALL.

## Key References

- Ribera J-M, Oriol A, Morgades M, Montesinos P, Sarrà J, González-Campos J, et al. Treatment of High-Risk Philadelphia Chromosome–Negative Acute Lymphoblastic Leukemia in Adolescents and Adults According to Early Cytologic Response and Minimal Residual Disease After Consolidation Assessed by Flow Cytometry: Final Results of the PETHEMA ALL-AR-03 Trial. Journal of Clinical Oncology. 2014;32 (15):1595 − 604.
  - This study illustrates the prognostic impact of minimal residual disease on relapse-free survival in ALL, incorporating MRD testing to guide risk-adapted therapy approach.
- Dhédin N, Huynh A, Maury S, Tabrizi R, Beldjord K, Asnafi V, et al. Role of allogeneic stem cell transplantation in adult patients with Ph-negative acute lymphoblastic leukemia. Blood. 2015;125 (16):2486-96.
  - This study shows for patients with high-risk Ph-negative ALL who achieve CR1, allo-HCT improves long-term survival and reduces relapse rates, namely in patients with detectable MRD post-induction.
- Litzow MR, Sun Z, Mattison RJ, Paietta EM, Roberts KG, Zhang Y, et al. Blinatumomab for MRD-Negative Acute Lymphoblastic Leukemia in Adults. N Engl J Med. 2024;391(4):320 − 33.
  - This study evaluates the use of blinatumomab in patients with Ph-negative ALL who are MRD-negative after induction therapy, providing significantly improved OS and RFS compared to conventional consolidation with chemotherapy alone.
- Kantarjian Hagop M, DeAngelo Daniel J, Stelljes M, Martinelli G, Liedtke M, Stock W, et al. Inotuzumab Ozogamicin versus Standard Therapy for Acute Lymphoblastic Leukemia. New England Journal of Medicine.375 (8):740 − 53.
  - This study highlights the safety, efficacy, and tolerability of inotuzumab ozogamicin in the consolidative setting for Ph-negative ALL.
- Roddie C, Sandhu Karamjeet S, Tholouli E, Logan Aaron C, Shaughnessy P, Barba P, et al. Obecabtagene Autoleucel in Adults with B-Cell Acute Lymphoblastic Leukemia. New England Journal of Medicine. 2024;391 (23):2219-30.
  - This study shows durable response and low rates of immune-related toxicities among patients with Ph-negative ALL who receive obecabtagene autoleucel.
- Bouchkouj N, Lin X, Wang X, Przepiorka D, Xu Z, Purohit-Sheth T, et al. FDA Approval Summary: Brexucabtagene Autoleucel for Treatment of Adults With Relapsed or Refractory B-Cell Precursor Acute Lymphoblastic Leukemia. Oncologist. 2022;27 (10):892-9.
  - This study offers evidence for the use of brexucabtagene autoleucel in Ph-negative ALL to achieve durable remissions and meaningful overall survival.
- Maude SL, Laetsch TW, Buechner J, Rives S, Boyer M, Bittencourt H, et al. Tisagenlecleucel in Children and Young Adults with B-Cell Lymphoblastic Leukemia. N Engl J Med. 2018;378 (5):439 − 48.
  - This study confirms the use of tisagenlecleucel in Ph-negative ALL to achieve sustained remissions with a favorable safety profile.

## Data Availability

No datasets were generated or analysed during the current study.
