# Correction: From MRD To Match: the Role of Allogeneic Hematopoietic Cell Transplant in Philadelphia-Negative B-ALL

**DOI:** 10.1007/s11899-025-00766-x

**Published:** 2025-12-04

**Authors:** Jessica El-Asmar, John C. Molina, Betty Ky Hamilton

**Affiliations:** 1 Department of Hematology and Medical Oncology, Taussig Cancer Institute, Cleveland Clinic, Cleveland, OH USA; 2 Leukemia Program, Department of Hematology and Medical Oncology, Taussig Cancer Institute, Cleveland Clinic, Cleveland, OH USA; 3 Blood and Marrow Transplant Program, Department of Hematology and Medical Oncology, Cleveland Clinic Cancer Institute, 9500 Euclid Avenue CA-60, Cleveland, OH 44195 USA

**Correction: Current Hematologic Malignancy Reports**

10.1007/s11899-025-00760-3

The original published version of this article unfortunately contained a mistake. Tables 1, 2, and 3 were inadvertently omitted and should have been included as shown below.

**Table 1:** Summary of high-risk features of Ph-negative B-cell ALL

**Cytogenetics**
• −7
• + 8
• t(4;11)
• t(8;14) or t(8;22)
• Intrachromosomal amplification of chromosome 2 (iAMP 21)
• Complex karyotype (5 or more chromosomal aberrations)
• Low hypodiploid (32–39 chromosomes)
• Near haploid (24–31 chromosomes)
**Molecular**
• *CRLF2* rearrangement
• *KMT2A* (MLL) rearrangement
• *IKZF1* deletion or loss-of-function mutations
• *TP53* mutation
• *JAK2* mutation
• *IDH1/2* mutations
**Measurable Residual Disease (MRD)**
• End of induction MRD ≥ 10^− 4^ by PCR or MFC
**Clinical Features**
• Age ≥ 40yo in adults
• Presenting white blood cell count ≥ 30 × 10^9^ /L
• Central nervous system involvement
• Time to CR1 > 4 weeks
• Requiring > 1 induction cycle
• Relapse beyond CR1

**Table 2:** Outcomes of allo-HCT among patients with Ph-negative ALL in CR1

**Study**	**Total Number of Patients**	**Number of Patients in CR1**	**Risk Stratification**	**Overall Survival (OS)**	**Clinically Relevant Outcomes**	**Comparative Arms**	**MRD**
**Huguet et al.** [24]	225	Not specified	Clinical and cytogenetic features	42-month OS: 60%	42-month EFS: 55%	Pediatric-Inspired Regimen	Not reported
**Dhédin et al.** [23]	522	282	Clinical features and post-induction MRD	Not specified	3-year RFS: 64.7% in allo-HCT arm NRM: 15.5%	Chemotherapy vs. allo-HCT	≥ 10^− 3^ by qPCR
**Zhang et al.** [15]	97	97	MRD status at CR1	3-year OS: 81.8%	3-year LFS: 81.8%	Chemotherapy vs. allo-HCT	10^− 4^ to 10^− 3^ by MFC
**Lv et al.** [20]	131	114	Clinical features, cytogenetics, MRD	2-year OS: 91.2%	2-year LFS: 80.9%	Chemotherapy vs. haploidentical allo-HCT	10^− 4^ to 10^− 3^ by MFC

**Table 3:** CAR T-cell products for ALL

	**Target Antigen**		**Co-Stimulatory Domain**	**FDA Approval (year)**	**Pivotal Trial (NCT)**	**Clinical Indication**	**Complete Response Rates**	**Grade ≥ 3 CRS (%)**	**Grade ≥ 3 ICANS (%)**	**Grade ≥ 3 Hematologic Toxicities**
**Brexucabtagene autoleucel (41)**		CD19	CD28	2021	ZUMA-3 (NCT02614066)	R/R B-ALL ≥ 18yo ≥ 1 prior lines of therapy	56–73%	24–26%	25–35%	• Neutropenia (90%) • Anemia (70%) • Thrombocytopenia (80%)
**Tisagenlecleucel (42)**		CD19	4-1BB	2017	ELIANA (NCT02435849)	R/R B-ALL ≤ 25yo ≥ 2 prior lines of therapy	60–81%	35–47%	13%	• Neutropenia (90%) • Anemia (70%) • Thrombocytopenia (80%)
**Obecabtagene autoleucel (39)**		CD19	4-1BB	2024	FELIX (NCT04404660)	R/R B-ALL ≥ 18yo ≥ 1 prior lines of therapy	55–77%	2–3%	7%	• Neutropenia (80%) • Anemia (60%) • Thrombocytopenia (70%)

The original article has been corrected.

